# QDs versus Alexa: reality of promising tools for immunocytochemistry

**DOI:** 10.1186/1477-3155-7-4

**Published:** 2009-05-27

**Authors:** Helena Montón, Carme Nogués, Emma Rossinyol, Onofre Castell, Mònica Roldán

**Affiliations:** 1 Servei de Microscòpia, Universitat Autònoma de Barcelona, Bellaterra Campus, 08193 Bellaterra, Barcelona, Spain; 2 Departament de Biologia Cellular, Fisiologia i Immunologia, Universitat Autònoma de Barcelona, Bellaterra Campus, 08193 Bellaterra, Barcelona, Spain

## Abstract

**Background:**

The unique photonic properties of the recently developed fluorescent semiconductor nanocrystals (QDs) have made them a potential tool in biological research. However, QDs are not yet a part of routine laboratory techniques. Double and triple immunocytochemistries were performed in HeLa cell cultures with commercial CdSe QDs conjugated to antibodies. The optical characteristics, due to which QDs can be used as immunolabels, were evaluated in terms of emission spectra, photostability and specificity.

**Results:**

QDs were used as secondary and tertiary antibodies to detect β-tubulin (microtubule network), GM130 (Golgi complex) and EEA1 (endosomal system). The data obtained were compared to homologous Alexa Fluor 594 organic dyes. It was found that QDs are excellent fluorochromes with higher intensity, narrower bandwidth values and higher photostability than Alexa dyes in an immunocytochemical process. In terms of specificity, QDs showed high specificity against GM130 and EEA1 primary antibodies, but poor specificity against β-tubulin. Alexa dyes showed good specificity for all the targets tested.

**Conclusion:**

This study demonstrates the great potential of QDs, as they are shown to have superior properties to Alexa dyes. Although their specificity still needs to be improved in some cases, QDs conjugated to antibodies can be used instead of organic molecules in routine immunocytochemistry.

## Background

Semiconductor nanocrystals called Quantum Dots (QDs) are fluorochromes with many advantages compared to the organic fluorescent dyes habitually used in immunocytochemistry procedures [1]. Their water solubility and capacity to be conjugated with different biomolecules have only recently been established [2]; therefore, their application in both the biological and medical research fields is still scarce.

Since the first microscope appeared up to the present day, different kinds of dyes (fluorescent proteins, small fluorescent molecules, etc.) have been used to detect or localize different biomolecules within an intracellular context. In the last decade, when nanotechnology became relevant, QDs were introduced as a promising methodological tool due to their intrinsic brightness, high photostability, high molar extinction coefficient, narrow emission band, and excitability with several wavelengths [3]. These qualities opened the possibility to handle samples labeled with different colors, preventing fluorescent signal crossing-over, using a single laser line to excite different QDs at the same time [4].

QDs are aggregates of atoms -from hundreds to tens of thousands that behave as one- of semiconductor materials that produce a crystalline matrix (nanocrystal). Composition, size and shape of this matrix determine their physical characteristics. The properties of nanocrystals vary according to their size, which ranges generally from 1 to 10 nm in diameter [5]; whereas smaller QDs emit in shorter wavelengths, bigger QDs emit in longer wavelengths. The crystalline core of QDs is composed of cadmium selenide and covered with a zinc sulfide shell. Moreover, some QDs are coated with different kinds of polymers and molecules in order to make them water-soluble and to facilitate their conjugation to different biomolecules, providing a specific functionality [6-9].

QDs can be linked to many molecules, such as DNA, proteins and antibodies, and therefore they have a wide range of applications in the biosciences. To date, QDs have been used to localize proteins [10,11] and mRNA within the cell [12], to label cancer markers [13], to follow in vivo metastatic cells during extravasation [14] or to track embryonic stem cells in deep tissues [15].

The aim of this study was to use QDs as secondary and tertiary antibodies in a routine immunocytochemistry procedure in which organic dyes are currently used. Therefore, we characterized the shape, size and optical properties of QD 655 (IgG or streptavidin conjugated) in order to develop a standard protocol for protein immunodetection using QDs. We have made a comparative study of fluorescence intensity, bandwidth, photostability, specificity and the quality of QD 655 versus its homologous organic fluorophore, Alexa 594 (IgG or streptavidin conjugated), to evaluate the possibility of replacing Alexa with QDs in this protein detection procedure.

## Results

### QDs characterization by HRTEM

**Figure 1:**
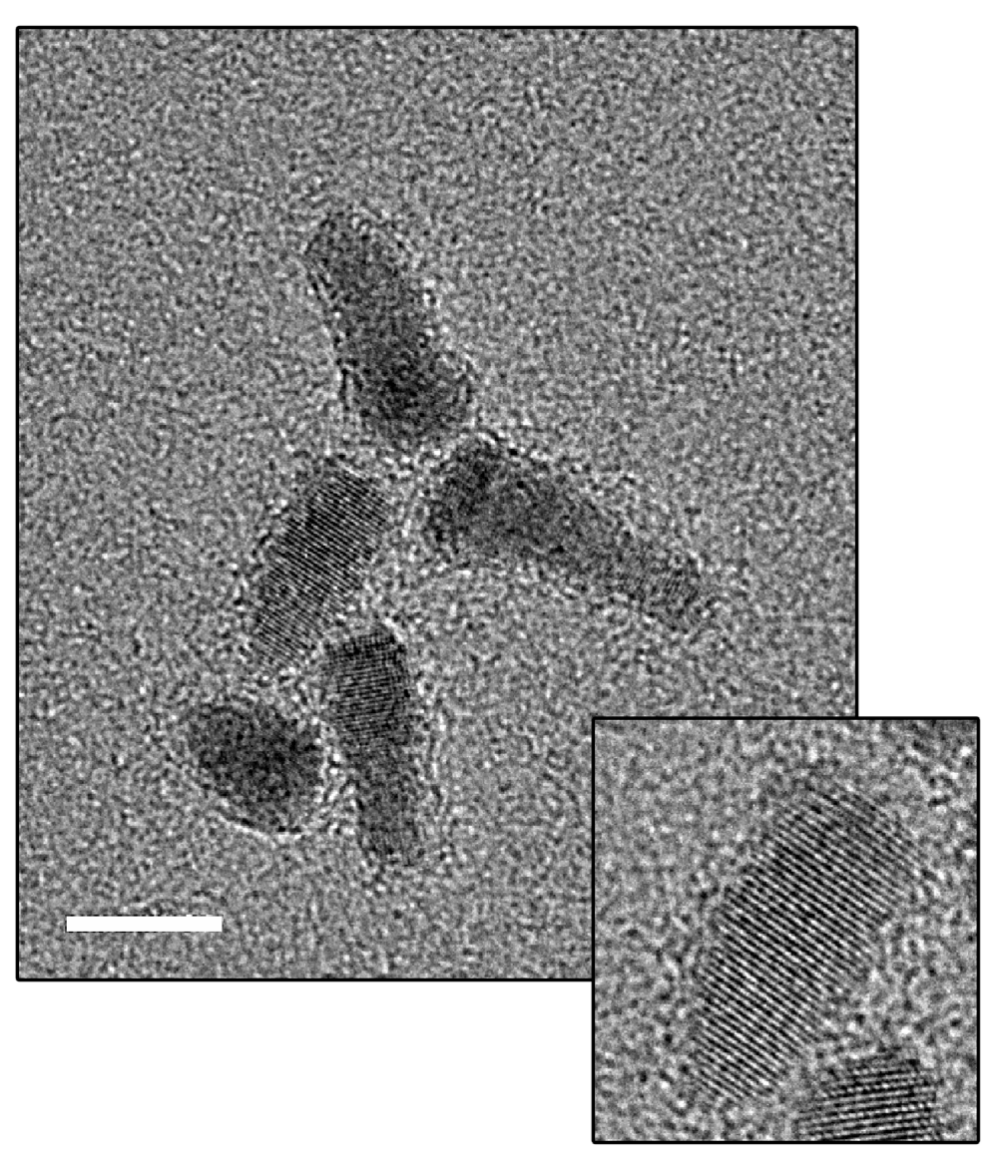
**HRTEM QD characterization**. The large image shows a general view of QD 655 dispersion. The small image shows a detail of a single QD 655 cone-like nanocrystal. Its crystalline structure core can be seen. Scale bar = 10 nm.

QD 655 showed a cone-like shape (Figure 1) with no differences in shape between QDs conjugated to streptavidin or to IgG. However, when comparing the size of the QDs conjugated to IgG with those conjugated to streptavidin, significant differences (p < 0.05) were found. QD 655-IgG is bigger (15.4 ± 0.2 × 6.4 ± 0.1 nm) than QD 655-strep (13.1 ± 2.8 × 6.3 ± 0.9 nm).

### QDs characterization by CLSM

QDs have been reported to present several optical advantages in fluorescence detection regarding conventional organic fluorophores. QD 655 has been compared to Alexa 594 to evaluate differences in fluorescence intensity, bandwidth, photostability and specificity.

#### Spectra emission

**Figure 2:**
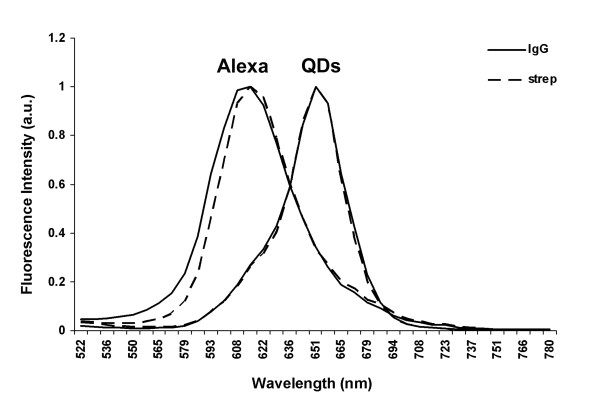
**Fluorescence emission spectra**. Spectral profile representing fluorescence intensity versus emission wavelength (500–780 nm) for QD 655-IgG, QD 655-Streptavidin and their Alexa homologues. Excitation wavelength = 488 nm.

First, the maximum fluorescence emission peak (λ_em_) of both fluorophores was assessed using the lambdascan function of the CLSM. QD 655 presented its maximum at 651 nm, whereas Alexa 594 had its peak at 615 nm (Figure 2, Table 1). The λ_em _value recorded was identical for the same fluorophore independently of its conjugation (IgG or strep).

**Table 1:** Spectral properties.

	Emission peak (nm)	FI^a^	Bandwidth (nm)^b^
Q655-IgG	651	200	35.5
Q655-strep	651	180	37
A594-IgG	615	75	48
A594-strep	615	75	53

Emission peak, fluorescence intensity (FI) and bandwidth for QD 655 and Alexa 594 analyzed at 488 nm excitation wavelength.

(a) Values in gray level (0–255).

(b) Calculated as the full width at 50% maximum of the emission spectrum (FWHM) in the FI profile.

Second, the fluorescence intensity (FI) level of QD 655 (Ig or Strep) and Alexa 594 (Ig or strep) was calculated. The FI level of QD 655 was higher than that of Alexa 594 (Table 1).

Differences in bandwidth, calculated from the emission profiles (Figure 2), were also found when both kinds of fluorophores were compared. QDs had narrower values of bandwidth than the homologous Alexas (Table 1).

#### Photostability

**Figure 3:**
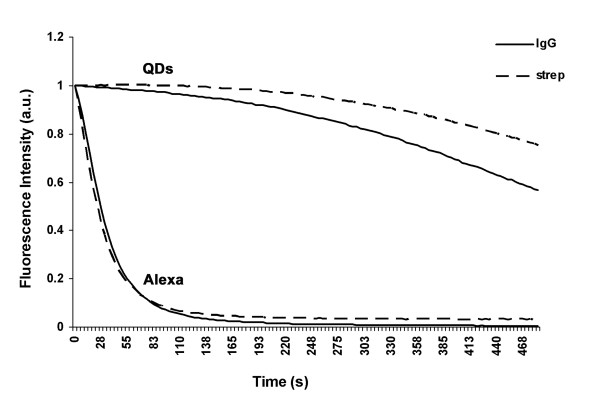
**Photostability profile**. Fluorescence intensity changes of QDs and Alexas during the irradiation period with the 561 nm laser line at maximum power.

**Figure 4:**
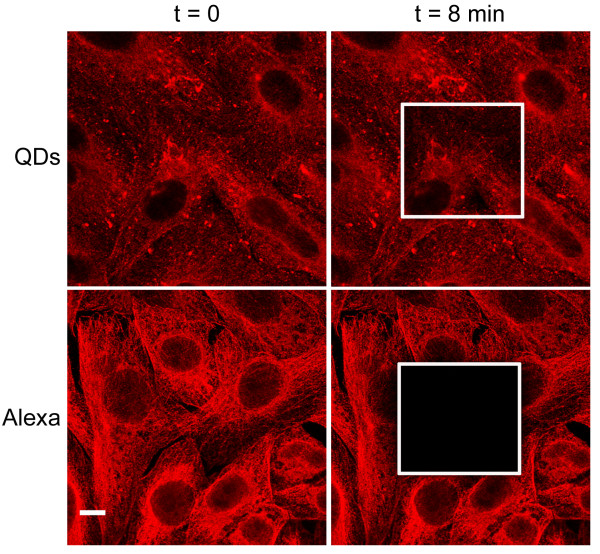
**CLSM photostability images**. Left-hand images correspond to the emission signal of QDs and Alexas conjugated to streptavidin before the irradiation period (t = 0 min) with the 561 nm laser line at its maximum power. Right-hand images show the emission signal of QDs and Alexas at the end of the irradiation period (t = 8 min). Note the loss of fluorescence intensity in the delimited area. Scale bar = 10 μm.

Photostability was assessed by exposing immunolabeled cultures for eight minutes at the maximum power laser line of 561 nm. For the first 90 seconds, the initial fluorescence intensity of β-tubulin labeled with QD 655s was reduced by about 5%. The same laser incidence produced an intensity reduction of 90% in cultures labeled with Alexa 594s. At the end of the irradiation period, no β-tubulin was detected in cultures labeled with Alexa 594s, whereas in cultures labeled with QD 655s, β-tubulin still kept up to 10%–40% of the initial fluorescence intensity (Figure 3 and 4).

#### Staining specificity

**Figure 5:**
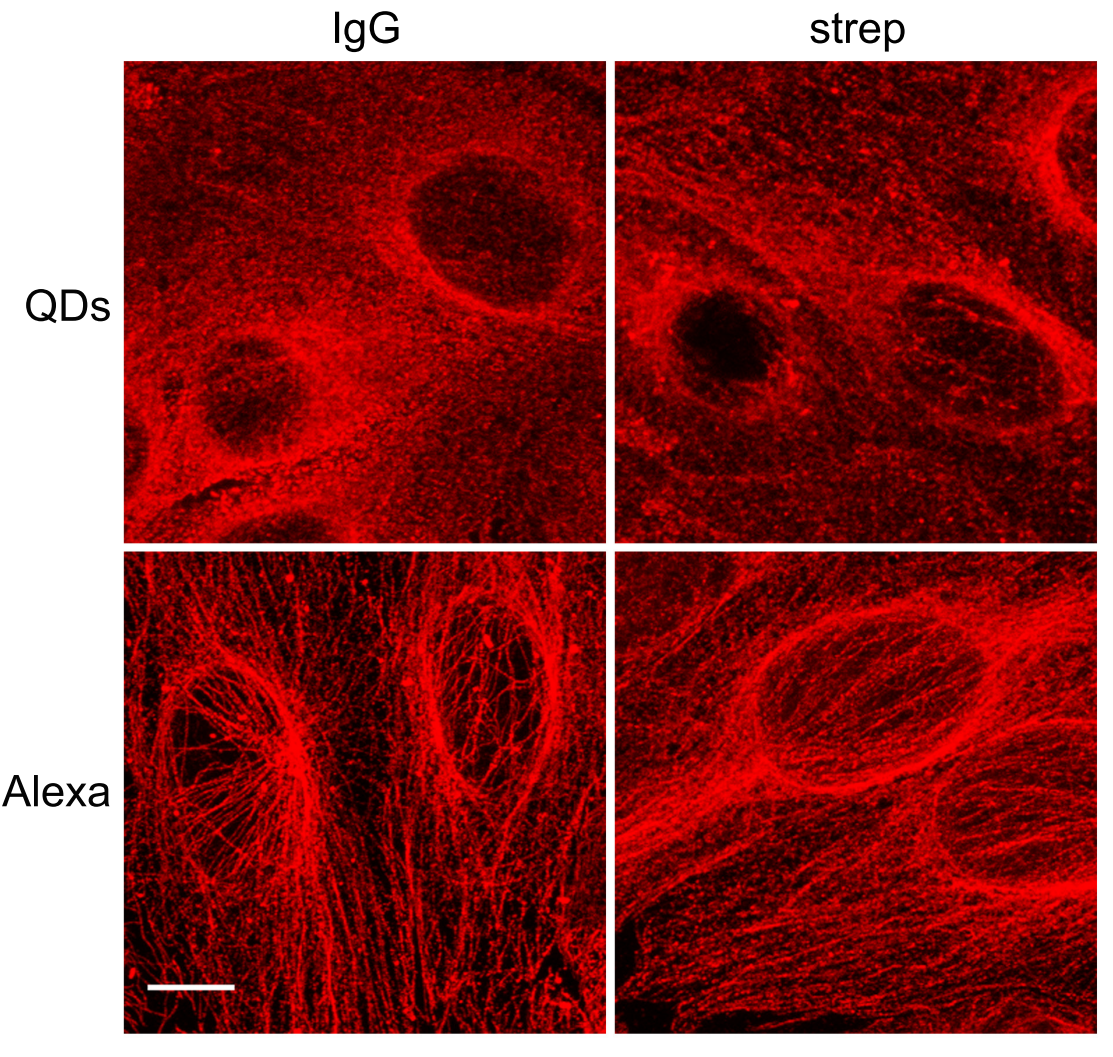
**CLSM specificity analysis of β-tubulin labeling**. Maximum intensity projections of the distribution of the tubulin network labeled with QDs (top images) show lower specificity than their organic Alexa homologue labeling (bottom images). Scale bar = 10 μm.

**Figure 6:**
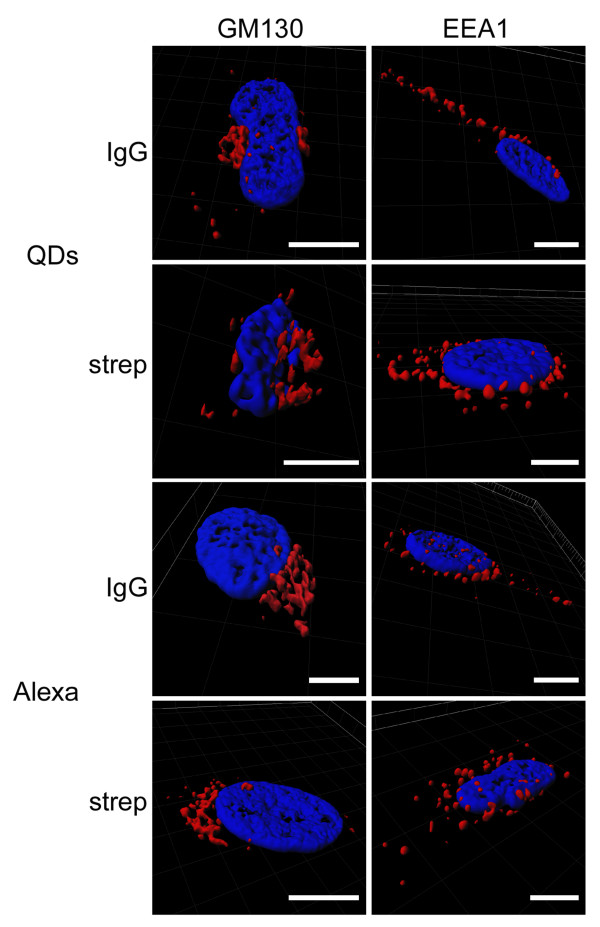
**CLSM specificity analysis of GM130 and EEA1 labeling**. Isosurface representation of the cell shows the nucleus (blue) labeled with Hoechst 33342, Golgi complex (GM130) and endosomal system (EEA1) (red) within a three-dimensional volumetric x-y-z data field. Scale bar = 10 μm.

Staining specificity was analyzed on cell cultures labeled with primary antibody against the microtubule network (β-tubulin), Golgi complex (GM130) or endosomal system (EEA1). Brightness of both fluorophores conjugated to IgG was similar (Figure 5). Differences in specificity were detected when QD 655-IgG was used as a secondary antibody against β-tubulin. The network of microtubules was not well defined, with background and QD aggregates that had not selectively linked to β-tubulin. In contrast, Alexa 594-IgG was very specific and the microtubule network was definitely detectable. When QDs and Alexas were used as tertiary antibodies, the tubulin network was clearly detected by both fluorophores, but Alexa fluorochromes were more specific in pinpointing the tubulin filament structure. No differences in specificity were detected when QD 655 or Alexa 594 was used as a secondary or tertiary antibody against GM130 or EEA1. Both types of fluorophores showed similar specificity (Figure 6).

## Discussion

In this work inorganic QDs were used to demonstrate their feasibility and advantages as a basic research technique in routine immunocytochemistry, as compared to Alexa organic dyes. To our knowledge, commercial QDs are not yet standardized; neither are they completely characterized to be used without further evaluation [16,17].

HRTEM characterization of QDs demonstrated differences in core size between the two types of QDs. In theory, these differences should be due to QD manufacturing, but the current methods used to produce QD allow particle size and particle size distribution to be controlled accurately [18]. Moreover, according to the Quantum Dot Corporation [2], there are only slight size differences in a given batch of QDs. However, other authors have found some variability in CdSe QDs size distribution [19].

One of the optical properties measured was the emission spectrum, which in QDs is related to their size. QD 655 conjugated to IgG or streptavidin displays a higher emission peak and a narrower bandwidth than its Alexa homologue. These advantageous characteristics have been well documented previously by different authors [4,13,20] and offer the possibility of using different QDs simultaneously without overlapping emission bands. The bandwidth of our batch of QD 655 (IgG and strep) was similar to that described in the literature [18,20].

Slight differences in size result in slight variations in the emission wavelength. As a consequence, the emission spectrum of a certain nanocrystal ensemble will be broader than an individual QD spectrum [21]. A variation in size distribution of 5% translates into a bandwidth of approximately 25–30 nm, a narrow value compared to the bandwidth of many fluorescent dyes [21]. Since the size distribution of each QD analyzed in this study was about 10%, it was expected that the bandwidth would be greater (ca. 35 nm).

Another optical characteristic analyzed was the intrinsic brightness of both fluorophores. The fluorescence intensity (FI) was higher in QDs than in Alexas. Most authors agree that QDs have superior brightness than organic fluorophores [1,13,20,22]. However, other studies have found that QDs are not as bright as expected [23]. Slight differences in FI (ca. 8%) were detected between both QDs, while in Alexas these differences were inappreciable. Other authors have found that the FI of QD 525-IgG was nearly one-third that of QD 525-streptavidin [24].

Photostability was the third optical property analyzed, and the entire scientific community agrees that this is the best advantage of QDs, as compared to other fluorescent dyes [3,5]. Our study confirms that QDs have the highest photostability. This characteristic is very important when in vivo analyses are carried out and long-term experiments are necessary and use multiple targets [25]. But photostability is also a determining factor in fixed samples in which some magnification is needed to find the best resolution to observe subcellular structures. Before QDs came out, Alexa dyes were considered to be the most photostable fluorophores [26]. Nowadays, this reality has changed: Alexa fluorophores lose almost all of their fluorescence in only 90 seconds of laser exposure, while we have demonstrated that QDs can be exposed to laser light for eight consecutive minutes and less than 40% of their initial fluorescence is lost.

All of these characteristics confirm that QDs have unique optical properties that make them powerful fluorescent dyes. In addition to the increasing interest in QDs in fluorescence techniques, their electron-dense core has potential to carry out correlated studies between CLSM and TEM, which would allow protein localization inside cells on a nanometric scale [11]. However, there is some controversy regarding the specificity of QDs as immunolabels. While some authors argue that QDs have comparable or even superior specificity in relation to organic fluorophores [27], others consider that QDs are appropriate fluorophores to be used as immunolabels, although without increasing sensitivity, and with higher, non-specific binding and aggregation than Alexa dyes [18,23]. Low specificity could be due to different reasons: i) a non-optimal concentration of QDs that could lead to a non-specific signal [1], or ii) a non-optimal surface chemistry of QDs that would affect their spectroscopic properties and colloidal stability as well as their biomolecular function or size, which could sterically hamper access to cellular targets [20]. Several authors have pointed out the importance of QD concentration for improving the sensitivity of detecting water pathogens [22], as well as improving specific immunostaining [1]. Before starting the QD characterization, we tested three different concentrations of QD 655 in order to use the most appropriate in which to perform this study (data not shown). The optimal concentration was 30 nM because there were scarce aggregates and the QD concentration was high enough to label the tubulin network.

On the other hand, specificity was higher when QDs were used as a tertiary antibody, but still lower compared to their Alexa homologue. Other authors have reported that QD sensibility is improved when they are used as tertiary antibodies [24]; this increase in sensibility is probably due to the high affinity between streptavidin and biotin, and to the signal amplification.

Finally, the specificity of QDs in detecting β-tubulin, GM130 and EEA1 proteins was tested. While specificity against β-tubulin was lower than Alexa, no differences were observed when QDs were used to stain the Golgi complex (GM130 protein) or endosomes (EEA1). Specificity of QDs was higher for primary antibodies against proteins like GM130 and EEA1, which are scarce in the cell and are not involved in the composition of thin structures. Specificity was lower for proteins such as β-tubulin which is an abundant protein in the cell and that polymerizes producing an extremely well organized thin structure. QD 655 is one of the largest QDs commercialized, and it is possible that its size could sterically obstruct its access to its target [20].

## Conclusion

QDs are excellent fluorophores for labeling cellular targets, as they display higher intensity, an enhanced signal to noise ratio, a narrower bandwidth and higher photostability than organic dyes. However, the specificity of QDs depends on the target they have to bind to. More studies are needed to improve the specificity of QDs so they can be used routinely, alone or in combination with organic fluorescent dyes, in all biological applications. In this study we were able to use QDs as secondary and tertiary antibodies to clearly detect discrete localized proteins. Therefore, in these cases, they can replace fluorescent organic molecules in routine immunocytochemistry procedures.

In the future, when better control of the synthesis and functionalization of QDs is possible, the range of biological applications of these fluorophores can be extended and they can become part of basic research techniques.

## Materials and methods

### Material

Two types of red emission spectra QDs were used as secondary and tertiary antibodies: QD 655 Goat F(ab)_2 _anti-mouse IgG conjugate (QD 655-IgG) and QD 655 streptavidin conjugate (QD 655-strep). Two homologous red emission Alexa Fluor Dyes: Alexa 594 Goat F(ab)_2 _anti-mouse IgG conjugate (Alexa 594-IgG) and Alexa 594 streptavidin conjugate (Alexa 594-strep) were used to compare to QD antibodies. Secondary antibodies QD 655-IgG and Alexa 594-IgG were purchased from Molecular Probes (Invitrogen Corp; Eugene, Oregon, USA), and Anti-Ms IgG biotin from Boheringer (Mannheim; Indianapolis, USA). Primary antibody monoclonal anti-β-tubulin was purchased from Sigma-Aldrich Chemie GmbH (Steinheim, Germany). GM130 and EEA1 primary antibodies were purchased from BD Biosciences (San Jose, California, USA).

### High Resolution Transmission Electron Microscopy (HRTEM)

To carry out a HRTEM analysis, 0.5 μl of each QD was diluted in 500 μl of MilliQ water and centrifuged for 10 minutes at 6000 rpm to eliminate all organic precipitates. A drop of each diluted QD was deposited on a carbon layer copper grid and air-dried.

Images of each type of QD were obtained with a HRTEM, using a JEOL JEM 2011 transmission electron microscope (Jeol LTD; Tokyo, Japan) operating at 200 kV. The sizes of the QDs were determined with Digital Micrograph software (Gatan Inc; Warrendale, Pennsylvania, USA) and data obtained were processed with statistics software Origin-8 (OriginLab Corporation; Northampton, Massachusetts, USA).

### Cell cultures

Two different culture cell lines, Vero (ATCC-CCL-81) and HeLa (ATCC-CCL-2), were used. Cells were maintained in MEM (GIBCO, Rockville, Maryland, USA) supplemented with 10% Fetal Calf Serum (GIBCO) and incubated at 37°C and 5% CO_2 _in a humidified atmosphere.

### Immunocytochemistry

For immunocytochemistry analysis, cells were seeded onto glass coverslips and incubated at 37°C and 5% CO2, until confluence was reached. Cells were fixed in 4% paraformaldehyde (Electron Microscopy Sciences; Fort Washington, Pennsylvania, USA) in 0.01 M phosphate buffer saline (Sigma Aldrich Chemie GmbH; Steinheim, Germany) for 15 min, permeabilized in 0.25% Triton X-100 (Fluka Chemie AG; Buchs, Switzerland) for 15 min and blocked in 6% bovine serum albumin (Sigma Aldrich Chemie GmbH; Steinheim, Germany) for 40 min. Finally, cells were incubated with the anti-β-tubulin monoclonal antibody (4 μg/ml) to detect the microtubule network, with the GM130 antibody (10 μg/ml) to detect the Golgi complex or with the EEA1 antibody (2.5 μg/ml) to detect the endosomal system. In all cases the primary antibody was incubated for 1 h at 37°C.

To perform secondary immunodetection, anti-Ms IgG antibody conjugated to Alexa or QDs (4 μg/ml and 30 nM final concentration, respectively) was used. For tertiary immunodetection, cells were first incubated with Anti-Ms IgG Biotin (1 μg/ml) for 1 h at 37°C, and then with streptavidin conjugated to Alexa or QDs (4 μg/ml and 30 nM final concentration, respectively). Coverslips were mounted onto glass slides using Fluoprep mounting media (bioMérieux^® ^SA, Marcy l'Etoile, France) to preserve fluorescence.

### Confocal Laser Scanning Microscopy (CLSM)

Images were captured with a CLSM Leica TCS-SP5 AOBS spectral (Leica Microsystems Heidelberg GmbH; Mannheim, Germany) using a Plan-Apochromatic 63× objective (NA 1.4, oil).

Series of images (xyλ), called lambdastacks, were taken to determine the spectra emission of QDs and Alexas and to establish their bandwidth. The excitation wavelength used was the 488 nm line of an Ar laser. The AOTF was set at 40% and 80% for QDs and Alexas, respectively.

The emission detection was set from 500 to 780 nm. The confocal pinhole for each lambdastack was fixed at 2 Airy units. For each xy focal plane, confocal microscopy measured the emission variation every 10 nm (lambda step size = 7 nm). The emission spectra analysis was processed using the CLSM software (Leica LAS AF). A Region of Interest (ROI) was delimited to determine the fluorescence intensity (FI) in the selected area in relation to the wavelength. To analyze immunolabeled cells, 45 ROIs of 2 μm^2 ^were selected near cell nuclei; FI and bandwidth were calculated in the selected ROIs.

Photostability experiments were performed using the Live Data Mode function of the CLSM, which permits monitoring long time-lapse experiments. Each type of fluorophore was illuminated with a 561 nm excitation laser line for 8 minutes (100% power, zoom = 6). Images were taken at 1 second intervals, in 512 × 512 pixels with 8 bits of dynamic range. In the area where the laser was at its maximum illumination power, 45 ROIs of 2 μm^2 ^were selected to show the FI in the region in relation to time.

Secondary or tertiary antibody specificity was evaluated using the xyz mode of the CLSM, which permits one to scan the xy plane along the z axis. Images were captured every 0.2 μm along 3 μm of thickness, with 1 Airy confocal pinhole. From the xyz series obtained by CLSM, maximum intensity projections were achieved with Leica LAS AF software, and three-dimensional models were generated using Imaris software (Bitplane; Zürich, Switzerland).

### Statistical analysis

To determine if there were significant differences in size between QDs conjugated to IgG or to streptavidin, a two-sample T-Student's test (T-test) for comparison of means, with 95% confidence, was carried out. Previously, a F-Fisher test was performed and equal variances were assumed due to the returned p-value of 0.389. The equality of means hypothesis was rejected when the p-value was lower than 0.05 (p < 0.05).

## Competing interests

The authors declare that they have no competing interests.

## Authors' contributions

HM performed the majority of the experiments and wrote the manuscript with MR and CN. ER contributed with the characterization by HRTEM and helped with data analysis. MR, CN and OC designed the overall project, helped with interpretation of data and revised the manuscript. All authors read and approved the final manuscript.
